# Lessons Learned: Occupational Therapy in Nursing Homes during the First Wave of COVID-19 in Spain

**DOI:** 10.3390/healthcare10010117

**Published:** 2022-01-07

**Authors:** Sara Chimento-Díaz, Isabel Espino-Tato, Jose M. Garcia-Alonso, Pablo A. Cantero-Garlito

**Affiliations:** 1 Department of Computer and Telematic Systems Engineering, Polytechnic School of Cáceres, University of Extremadura, 10003 Cáceres, Spain; isabelet@unex.es (I.E.-T.); jgaralo@unex.es (J.M.G.-A.); 2 Faculty of Health Sciences, University of Castilla-La Mancha, 45600 Talavera de la Reina, Spain; Pablo.Cantero@uclm.es

**Keywords:** pandemic, coronavirus, nursing homes, occupational therapy

## Abstract

This research aims to explore the perception that occupational therapists working in elderly care facilities have about the measures implemented against the COVID-19 pandemic in their resources, and the impact that these measures have had on occupational therapy in these facilities. An interpretive paradigm was selected, using a qualitative approach and a phenomenological design. Sixteen occupational therapists working in elderly care facilities in two Spanish regions were included. Data were collected through semi-structured interviews. A discourse analysis of the narrative information was carried out using open, axial, and selective coding processes and the constant comparison method. Four themes were extracted from the analysis results: The initial chaos in senior centers; The blurring of occupational therapists’ roles; The emergence of technology; and organizational and therapeutic proposals for future pandemics. The pandemic had a significant impact on the care and therapeutic processes in elderly care facilities. Occupational therapists had to stop performing their functions to dedicate themselves to other support, auxiliary or communication tasks between the center and the families. Similarly, it is worth noting the emergence of low-cost technology to facilitate communication and to carry out some therapeutic interventions.

## 1. Introduction

The outbreak and rapid international spread of the severe acute respiratory syndrome coronavirus (SARS-CoV-2), responsible for COVID-19 [1,2], has become a public health emergency that has causedmany cases worldwide and thousands of deaths. Spain has been one of the countries where the pandemic has been most virulent, causing at the time of writing (October 2021) nearly 5 million cases and 87,030 deaths, one of the highest figures worldwide. Since the beginning of the COVID-19 pandemic, it was known that people over 70 years of age, especially those living in residential centers, had a much higher risk of death than those living in other contexts [3]. In Spain, the first wave of COVID-19 was particularly virulent, which forced the health system to implement drastic strategies to deal with the pandemic that radically changed the daily lives of millions of people from one day to the next. The measures developed both in many homes for the elderly and in many of the care facilities for people with disabilities have meant that residents spent a significant number of hours a day in their rooms, that stimulation, rehabilitation, and care activities were significantly reduced, or even that many facilities saw their doors closed. Moreover, the interventions that had been carried out up to that time were now carried out virtually [4]. Occupational therapy has played a leading role in nursing homes since the 1960s. The discipline’s conceptualization of subjects as occupational beings hasaddressedthe needs of older adults living in residential facilities. Participation in occupations has been associated with increased quality of life andincreased life satisfaction [5]. During the COVID-19 pandemic, occupational therapy in this type of resource has had to adapt urgently and essentially, betting on interventions based on telehealth and telerehabilitation or making the tasks to be performed by professionals more flexible [6,7]. The present study explores the perception that occupational therapists working in care facilities for the elderly have about the measures implemented against the COVID-19 pandemic in their facilities. Furthermore, more specifically, we aim to understant the impact of prevention measures against COVID-19 on the development of occupational therapy in elderly care centers.

## 2. Materials and Methods

### 2.1. Study Design

We used a qualitative approach to carry out this study, following a phenomenological design [8]. This design focuses on the commonality of a lived experience within a given group. Therefore, this allows for a better description of how people understand and comprehend certain phenomena. Furthermore, uniqueness of this perspective allows for a description of the essence of the experience for all participants; i.e., “what” they experienced and “how” they experienced it [9].

### 2.2. Participants

We selected participants between March and May 2021, following a purposive theoretical sampling. The inclusion criteria were: being or having been part of a work team of residential centers or day centers during the months March to June 2020, i.e., during the first months of confinement caused by the health crisis provoked by COVID-19. The final sample included 16 participants (average 30 years). A total of 16 interviews were carried out, until data saturation, from the autonomous communities of Extremadura (9) and Castilla La Mancha (7). The recruitment strategy consisted of contacting different occupational therapists in the regions of Extremadura and Castilla–La Mancha to explain the study’s aim and arrange an appointment at their convenience. Once the appointment was made, the purpose of the interview was explained again, and information about the study was provided in writing. Their main sociodemographic characteristics are shown in (Table 1).

### 2.3. Data Collection

An in-depth interview was used to collect data [10] guided by a series of semi-structured questions, although with the possibility of exploring and going deeper into other topics that the researchers had not initially considered but that arose during the interview [11]. The interview was focused on knowing the action protocol carried out in the residential centers and the role played by the occupational therapy professional and the discipline itself. All interviews were conducted between March and May 2021. These were conducted through different video call applications such as Zoom or Teams [12,13] to adequately comply with the prevention measures established by the health authorities. The initial interview script was based on the objective of the study, following other related work conducted in other settings or other similar contexts (Table 2). The interview questions were subsequently modified to adapt to the contents emerging from the participants’ accounts. These interviews were conducted by two of the researchers (SCD and PACG), both with extensive experience in qualitative research and in the use of in-depth interviews.

Data were collected from participants through in-depth interviews. The duration of the in-depth interviews was between 30 and 60 min.The investigators agreed to terminate data collection when data saturation was reached [14]. To ensure anonymity, we removed all participants’ characteristics from this article. Once the interviews were transcribed, the original audio recordings were destroyed.

### 2.4. Data Analysis

The recorded interviews were transcribed and subsequently analyzed using WebQDA software.WebQDA is a software created to support the qualitative dates analyses in a collaborative and distributed work environment such as the Internet synchronously or asynchronously [15]. The participants’ accounts were analyzed thematically. The various research team members met periodically throughout the study to analyze and review the data obtained. The participants’ accounts were initially coded by one of the researcher (IET) following an inductive approach. The discourse analysis process was carried out according to the following sequence. Once the units were established, an open coding process was carried out, following a constant comparison procedure [16]. Then, through an axial coding process [8], the subcategories were integrated into broader categories. Finally, the categories were grouped into three themes corresponding to the study’s objectives.

### 2.5. Methodological Rigour Criteria

To ensure the quality of the study, we followed the guidelines of the Consolidated Criteria for Reporting Qualitative Research (COREQ) [17].

### 2.6. Ethical Considerations

These research procedures were conducted following the bioethical principles set out in the Belmont Report, together with the Declaration of Helsinki and the Convention on Human Rights and Biomedicine of the Council of Europe. All participants gave informed consent. Confidentiality was maintained throughout the study, and only the research team has had access to the data obtained. All information generated and collected during this study complies with the Organic Law 3/2018 of 5 December on the Protection of Personal Data. The study was approved by the Bioethics and Biosafety Commission of the University of Extremadura (81//2021).

## 3. Results

The results of the content analysis of the interviews have yielded four themes that describe how occupational therapists carried out their work during the first wave of the COVID-19 pandemic. The discovered themes were: (1) Initial chaos in seniors centers; (2) The blurring of occupational therapist´s functions; (3) The emergence of the technology and (4) Changing the system: organizational and therapeutic proposals for future pandemics.

### 3.1. Initial Chaos in Senior Centers

The participants in the study reported that at the beginning of the pandemic, all the centers were involved in initial chaos due to the lack of information about the situation that had arisen. The lack of knowledge about the virus, the ways that it spread, and the general confusion led, to a certain extent, to a general feeling of insecurity on the part of the healthcare team in the center:

*“The staff felt very anxious. You did not know which way to turn. It was, as I said at the beginning, chaos. Nothing was clear to you. You saw people getting worse and dying, and you saw the ambulance or the funeral parlor at the door, and in the morning and you left and came back.”* (TO6)

One of the first measures taken as soon as the state of alarm was imposed was to restrict visits and close the centers, although some of them, put on notice by their nurses or doctors, decided a few days earlier not to allow access to all those who were not workers of the center:

*“We had a meeting prior to the state of alarm where it is true that the doctor of the residence put us on prior notice of what was to come and we closed.”* (TO10)

Regarding the prevention measures that were carried out, one of the measures that gained more value and that most of the interviewees shared were the creation of bubble groups among employees to reduce contact and thus always work with the same colleagues and avoid the spread, as well as the immediate restriction of visits and access of people outside the center.

*“We created bubble groups so as not to mix and not to have too many people in the same room, so the physio and I would agree, if she worked with five people in the gym, then those same people would go to therapy with me.”* (TO16)

*“The first thing they did was to close all visits to the center. Only the staff working at the time could enter; in fact, some of the female colleagues were even asked to stay in the center.”* (TO12)

*“Well, first of all, they closed the exit of the residents to the outside and the visitors could not enter the center except for the workers.”* (TO10)

Another of the measures that were taken and that have already been established as a protocol was to have complete control of the body temperature and vital signs of resident, and, after the reopening, also to visitors and people outside the center. Likewise, all the centers added to these measures the use of specific material such as masks, PPE (personal protective equipment), double gloves, screens, goggles.

*“Well, they checked the temperature with the thermometer daily.”* (TO14)

*“As a maximum precaution, everyone in residence, including me, among many others in terms of material, use PPE, we use a hat, a mask and gloves, we have to wash our uniforms daily, and at high temperature in that sense. In terms of PPE, we had extreme measures.”* (TO11)

### 3.2. The Blurring of Occupational Therapist’s Functions

One of the questions that generated the most interest in the study was to know what role you have played as occupational therapists throughout this process. As a result of the pandemic, we concluded that occupational therapists had changed their role within nursing homes. Some respondents report reorganizing their work, either by doing it online, reducing intervention groups, or suspending activities to take on new roles in the team and help with tasks that required special attention.

*“Well, I had to reorganize the session a little bit in groups and so on, but during the pandemic, it was not altered, and my activities it is true that I, for example, did them as a notebook.”* (TO16)

The suspension of occupational therapy activities during the pandemic has been relatively standard, either due to lack of time or lack of staff, since, as mentioned above, many people had to act as reinforcement and support, in terms of enabling “COVID plans”, participate in standard room adaptation processes, enforcing safety protocols

*“I did not do my work as such. The therapies were suspended, the rehabilitation was suspended except in specific cases because, of course, it took us about three hours to get up and give breakfast to everyone. And when you were finished at that time, you had to start feeding them, room by room.”* (TO1)

*“In the beginning, it was a bit like I don’t do what I have to do, and it is a bit weird because you do not see yourself useful in the center, but because your work now has to focus on other things.”* (TO14)

*“My day was to make calls and get them [residents] to eat.”* (TO3)

*“I had to do all the functions of sending daily emails and ordering the material.”* (TO7)

Out of the total number of respondents, only one of the centers has continued to provide occupational therapy sessions.

*“Yes, of course, in addition, everyone has their individualized notebook. They are given the material they need. We give each one a box with the material. The material with the activities they had to do and everything you know very well organized.”* (TO13)

Due to this change in responsibilities, occupational therapists have put the role of occupational therapy on the back burner in most centers. Some have decided to restructure their sessions and make them individualize, and the minority of centers have continued to use occupation as a fundamental pillar. In some cases, a strange sensation of well-being was felt since the time dedicated to the person was of quality and individualized for each resident.

*“We had to reinvent our work, look for another working methodology, because before we had group sessions, but those group sessions were radically eliminated. We used to work in the swimming pool and the gym, and I in the occupational therapy room, and this work was moved to the rooms because the residents were confined to the rooms for a while, so that is hard for them”.* (TO10)

### 3.3. The Emergence of Technology

One of the functions that many professionals had to perform was to act as a link between user and family, so it was necessary to know the role of technology during the pandemic. In this case, most of the participating centers acknowledged that their use of technology has changed during these months, from the occasional phone call to using all kinds of devices to make video calls almost every day.

*“The truth is that it has played an important role because it has been the only means of communication that we could have.”* (TO15)

*“Very important, very important. They have been the only ones who have kept him in contact with his family. They were able to see his face and his family.”* (TO5)

During these months, the most used technological device has been the Tablet, followed by cell phones.

*“We bought a lot of tablets.”* (TO2)

Some respondents refer to the importance of the use of new technologies, stating that they are here to stay. On the contrary, only two of the centers stated that they do not believe there will be a change in their use of them shortly.

*“Yes, they will stay because I think they are a helpful tool. I already believed it before, when I used it in therapy with a Wii [home console].”* (TO4)

### 3.4. Changing the System: Organizational and Therapeutic Proposals for Future Pandemics

To the question “What could we change to improve the system?” it could be observed that most of the centers have stated that several of the measures imposed by the government during this time could be improved. The most critical point has been the time of publication between the different protocols and how difficult it has been for them to have to read them before entering that same morning to work and having to change everything that was being done from one day to the next, without to take into account the demands of the users and the workload of all the professionals.

*“I think that was the main thing because you cannot make the protocol and publish it in the afternoon and at 10 a.m. the next day send me another protocol because, of course, that in the end is a waste of time and resources in itself…”* (TO16)

The lack of communication they have had with the administration and public health has become evident. Additionally, there has been a lack of personnel necessary to carry out all the protocols correctly.

*”I think we have been very lost, they did not answer us, we had a positive case in the center, and Public Health said that whatever we wanted to do.“* (TO9)

*”Sure, and more staff, especially. However, I did not have to give up my occupational therapy duties to take on other management duties while the director was away because she was on leave.“* (TO7)

Finally, after primarily overcoming one of the most extreme situations in recent decades, we asked the people surveyed: What have we learned from all this? Making a general review of everything that has happened to us over the last year, the professionals of these centers affirm that they have changed both professionally and personally. Many of them agree that they have learned to value the little things in life that we have as a routine and that perhaps we put aside and do not give them importance.

*”At a personal work level, the importance of those little things that we do not pay enough attention to, that is, that routine of social contact, which as you were saying, I was not so affectionate before and even now, I miss it.“* (TO11)

*”I think we have learned to value things like maybe the routine and the people that we see every day and that we think are essentia, but we did not realize that maybe having them every day does not mean that they will always be there“* (TO12)

From a professional point of view, respondents have learned to focus more on people and what they need in each moment rather than on finishing a session designed for today. At the same time, they also certify having learned to organize their day-to-day life better and adapt to a new situation without generating stress.

*”Well, I have learned to manage everything in an organized way and to try to manage the fear of uncertainty“.* (TO15)

Finally, some of the respondents explained that as a result of the pandemic, they have realized that the general population has given more importance to health personnel and that thanks to occupational therapists, they have been able to see the need and importance of occupation in our daily lives.

*”in a moment like when they take away all the occupation in the world is when you realize how vital occupation is.* (TO7)

*“I think we have been one of the people who have been more flexible in everything because we have adapted as much as we can. Our role is so broad that we have many possibilities.”* (TO6)

## 4. Discussion

After analyzing the results, and how we could see at study by Laviero and colleagues [18], the figure of occupational therapists has been vital, being considered as flexible labor staff since they have been subjected to changes in terms of their functions and the organization of their sessions. Some occupational therapists stated that they have had to suspend their occupational therapy sessions to perform different functions outside their responsibilities, such as management or coordination tasks, due to the lack of staff in the centers. They have also had to give breakfast to residents, help colleagues and, above all, take charge of daily telephone contact between users and their families. In addition, we can observe that, as it is stated, being able to work through a technological device like tablet or smartphones from occupational therapy has marked a turning point in the relationship between professionals and residents’ families. On the other hand, although to a lesser extent, there were professionals who were able to maintain and carry out their functions and the schedule of their sessions during the first months of confinement. However, they differed throughout this time, and they had to adapt to the new situation and reschedule all their activities individually since the users were isolated in their rooms. As asserted in the study by Flores-Tena [19], the pandemic has demonstrated the relevance of the occupational therapist in the strategic development of good occupational practice for their users. Most of the people interviewed have emphasized how the decline in the residents’ physical and cognitive abilities has become evident, despite having had access to individual rehabilitation sessions. Interpreting the results, we can observe that technology has played a crucial role during this process for occupational therapists. Although the most commonly used devices for family contact in most centers have been Tablets and smartphones, different therapists have gradually integrated these devices into their daily treatment sessions. As a result of the isolation situation, it has been observed how it has been the residents themselves who have asked their relatives to have their smartphone to maintain contact since in recent months, social networks and communication has increased so as not to feel isolated during the pandemic. The same happened in Chimento and colleagues [20], where they observed differences between if people were in your home during the first months of a pandemic or if the people were living a residence. One of the factors they referred to was the fear of loneliness and not having contact with family members in case the confinement situation was repeated. The use of technological devices and even virtual rehabilitation sessions can have numerous benefits, such as eliminating the barriers that geographical distances imply. In addition, it has been necessary for the family members themselves to see from another perspective the work being done to intervene and collaborate in the process. On the contrary, some centers have not considered technology adequate and have decided to maintain the supervised telephone call with family members exclusively since the residents found the use of technology complicated. All this reflects that the social support network in this vulnerable group of the population becomes weaker, causing them to have even fewer social relationships and decreasing their social participation in the community or the development of recreational activities. Furthermore, the guidelines that have been carried out in the centers as security protocols have not favored the situation and, in most cases, has led to a a detriment of both cognitive and physical abilities of most of the residents. One of the first measures taken as soon as the state of alarm was imposed was to restrict visits and close the centers, although some of them, put on notice by their nurses or doctors, decided a few days earlier not to allow access to all those who were not workers of the center. Moreover, as we observe at the Official Gazette of the State of 21 March [21] during these months of the pandemic, it has been essential for the centers to monitor the vital signs of all their residents more exhaustively since one of the most frequent symptoms presented by those infected is fever. Another of the new measures that have been implemented in recent months has been the creation of stable coexistence groups known as “bubble groups,” a key plan to keep possible contagions under control and prevent the spread. One aspect perceived as detrimental that derived from this situation was the general exhaustion of both the support figures and the residents as the period of isolation was extended. In addition, many of the professionals initially found it hard to adapt to this change because they had to restructure the mode of intervention, from teaching new protocols to the use of technology. However, for users, one of the aspects that was perceived as being facilitating was not having to travel to the occupational therapy session. In addition, some professionals perceived that individual sessions could be more effective with some users when addressing the objectives, as they allowed you to emphasize the needs and demands of each one. Finally, an overall assessment of the situation was made, trying to draw positive aspects from all experiences in the last months and all they had learned as professionals. Most of them affirmed that all this context had managed to give more visibility to the work of occupational therapists and the importance of occupation in our daily lives. Nevertheless, on the other hand, some occupational therapy professionals referred to how we now value the little things in our lives that we consider will always be there and to which we do not give importance and that we had lost during these months. Within the limitations of this work, it is worth considering the differences in size of the occupational therapists’ residences, which could influence how they perceive their experiences in different ways. On the other hand, the interviews were conducted one year after the onset of the pandemic. This fact could influence both personal and professional recollection of the impact of the measures implemented to deal with COVID-19. As regards future lines of research, it would be interesting to carry out studies that would delve deeper into the measures that have been implemented subsequently and how these have changed the way of organizing both the residences in general and, more specifically, the work carried out by occupational therapy.

## 5. Conclusions

The outbreak of the Covid-19 pandemic had a significant impact on the care and therapeutic processes in the core resources for the elderly. This meant that in most cases, occupational therapists had to stop performing the specific functions of the profession to devote themselves to supporting others, and perform auxiliary or communication tasks between the center and the families. Similarly, it is worth mentioning the emergence of low-cost technology to facilitate communication and carry out therapeutic interventions. The need for procedures that provide the occupational therapist with information, higher levels of protection against stress, and measures that favor the development of occupational therapy in circumstances of isolation and confinement is highlighted.

## Figures and Tables

**Table 1:** Main Sociodemographic Characteristics.

Participant	Sex	Age	Management Type	Center Size	Type of Population
TO1	Man	33	Concerted	100	Rural
TO2	Woman	26	Concerted	130	Rural
TO3	Woman	30	Private	196	Urban
TO4	Woman	41	Concerted	108	Rural
TO5	Woman	30	Concerted	100	Urban
TO6	Man	45	Concerted	110	Rural
TO7	Woman	27	Concerted	100	Rural
TO8	Woman	31	Concerted	140	Urban
TO9	Woman	32	Private	50	Urban
TO10	Woman	26	Concerted	140	Urban
TO11	Woman	25	Concerted	150	Urban
TO12	Woman	24	Concerted	98	Rural
TO13	Woman	30	Private	60	Rural
TO14	Woman	34	Private	110	Urban
TO15	Woman	24	Concerted	30	Urban
TO16	Woman	25	Private	70	Rural

**Table 2:** Semi-structured interview script.

Questions
What role has technology played?
Has the use of technology changed from how it was used before and how it is used now?
How do you think you have been influenced by the change in social perception of people who have worked with the most at-risk population?
What role have you played as an occupational therapist during the first weeks of the pandemic?
What role has occupational therapy played throughout the process?
What would need to be changed to improve the system?
What can be learned about what has happened over this year?

## Data Availability

Not applicable.
